# Role of HEG1 and Claudin-4 Immunohistochemistry in the Differential Diagnosis of Lung Adenocarcinoma from Malignant Mesothelioma in Pleural Effusion Cytology

**DOI:** 10.5146/tjpath.2025.13801

**Published:** 2025-05-31

**Authors:** Aziza E. Abdelrahman, Fatma A. Elbadry, Taiseer R. Ibrahim, Mohamed Ali Alabiad, Mohamed Awad

**Affiliations:** Department of Pathology, Faculty of Medicine, Zagazig University, Zagazig, Egypt; Department of Pathology, Faculty of Medicine, Zagazig University, Zagazig, Egypt; Department of Pathology, General Medicine Practice Program, Batterjee Medical College, Jeddah, Saudi Arabia; SHO, Department of General Surgery, Bedford Hospital NHS Foundation Trust, Bedford, UK

**Keywords:** HEG1, Claudin-4, Malignant mesothelioma, Lung adenocarcinoma, Pleural effusion

## Abstract

***Objective: *** Cytological examination of pleural effusion is minimally invasive and low risk but faces challenges due to the lack of architectural features, low cell counts, and overlapping characteristics among reactive mesothelial cells (RMCs), carcinoma cells, and malignant epithelioid mesothelioma (MPM) cells.

The aim of this was study to detect the diagnostic accuracy of the expression of HEG1 and Claudin-4 in distinguishing malignant mesothelioma from lung adenocarcinoma in pleural effusion.

***Material and Methods:*** The present study was carried out on 84 cases of pleural effusion. Sixty-four representative cell blocks of the studied malignant cases and twenty control cases were stained with HEG1 and Claudin-4 immunostaining, and the results were recorded.

***Results: *** Positive membranous HEG1 immunoexpression was found in 95% of RMCs in benign effusions. Also, positive membranous HEG1 immunoexpression was found in 96% of cases of MPM, and only 2.6% of lung adenocarcinoma cases. There was a statistically significant difference between benign effusion with RMCs and lung adenocarcinoma immunoreactivity. There was a highly statistically significant difference between HEG1 immunoexpression in MPM and lung adenocarcinoma. On the other hand, all cases of benign effusions and all MPM cases had negative Claudin-4 immunoexpression while positive membranous Claudin-4 immunoexpression was found in 94.9% of lung adenocarcinoma cases. There was a statistically significant difference in immunoexpression of Claudin-4 between benign effusion and lung adenocarcinoma. There was a statistically significant difference in the immunoexpression of Claudin-4 between MPM and lung adenocarcinoma.

***Conclusion:*** HEG1 and Claudin-4 IHC staining is extremely valuable in the differential diagnosis between reactive or malignant mesothelial cells and adenocarcinoma in pleural effusion.

## INTRODUCTION

The sensitivity of conventional pleural effusion cytology alone to diagnose malignant pleural mesothelioma (MPM) varies greatly, from 16% to 73%. Pleural effusion patients can avoid more intrusive tests to obtain a tissue sample, which reduces the possibility of procedural issues and also saves costs and time (1).

Because they desquamate quickly, adenocarcinomas have the best accuracy rate for cytology in malignant pleural effusions (MPE). The cytopathologist’s interest and expertise level, the preparation technique, and the sample quality all affect how sensitive the cytological diagnosis of pleural fluid is. Thus, in patients with good functional status, thoracoscopy combined with pleural biopsy is still the gold standard for diagnosing MPE (2).

In addition, a high concentration of inflammatory cells or heavy blood staining may lessen the number of cells in effusion specimens. Concentration methods that improve the detection of cancerous cells can get around these issues, such are preparations for cytospin and cell blocks. Additionally, cell blocks can serve as a substrate for the application of immunocytochemistry (ICC) and molecular procedures, among other adjunctive assays (3).

Experience as a cytopathologist is another crucial factor, since cytopathology is a recognized subspecialty in and of itself. For instance, malignant and benign reactive mesothelial cells (RMCs) might resemble each other morphologically, making the diagnosis more difficult and requiring careful evaluation. One such potential restriction could be the amount of pleural effusion that was submitted for analysis (4).

It is advised to use an immunohistochemistry (IHC) panel in the diagnostic workup for the differential diagnosis of MPM with epithelioid morphology and metastatic mimicking carcinomas, containing a minimum of two mesothelial markers and two pancarcinoma markers. However, no gold standard has been created, and a number of antibodies and panels with varying sensitivity and specificity have been documented in the literature (5).

The aim of this study was to ascertain the diagnostic accuracy (specificity and sensitivity) of HEG1 and Claudin-4 expression in differentiating between lung adenocarcinoma and malignant mesothelioma in pleural effusion cases.

## MATERIALS and METHODS

Following approval (IRB#:905-24-10-2021) from the institutional review board (I.R.B.) we collected 84 pleural effusion samples from the departments of chest and cardiothoracic surgery (64 malignant pleural effusions and 20 benign effusions) between December 2021 and May 2023. Complete clinical data, including age, sex, clinical presentation, radiological examinations, and the physical appearance of each pleural effusion specimen were collected after obtaining the patient’s medical files. Pleural aspirates were subjected to routine cytological analysis using H&E-stained smears, and cell blocks were prepared from the sediment that was centrifuged from all the samples.

The study comprised cases with enough cells in cytology and cell blocks that were classified as lung adenocarcinoma, MPM, or RMCs with complete clinical and radiological data.

We excluded cases with secondary lung cancer, primary lung cancer other than adenocarcinoma or MPM, lung biopsy without pleural effusion, pleural effusion without biopsy sample, insufficient or non-representative samples, and suspicious cases with uncertain diagnosis.

### Cytopathological Study

#### 1. Cytological smear preparation

The pleural fluid samples were aspirated, tapered into centrifuge tubes, and spun for ten minutes at 2000 rpm. After centrifugation, the sediment was treated to create cell blocks.

#### 2. Cell-block preparation

Samples were regularly fixed in neutral buffered formalin 10% before being treated in paraffin to create cell blocks. Four µm thick slices were created for each cell block. Histological analysis was done using H&E staining, while the other sections were immunostained with HEG1 and Claudin-4.

#### 3. Cytological and cell block evaluation

Cytological smears and H&E-stained parts of cell blocks were evaluated. The results of the cytological investigation were categorized as unsatisfactory, negative, positive, or suspected of cancer based on morphological criteria. To ascertain the cellularity of the cases selected for the study and to confirm the diagnosis, cell block sections were evaluated.

### Immunocytochemical study

Sixty-four representative cell blocks of the malignant patients under investigation and twenty control cases were immunostained with HEG1 and Claudin-4, and the results were recorded.

### Immunocytochemical stains

Using a polymer detecting system, immunocytochemical (ICC) staining was performed utilizing the following:** **HEG1: Rabbit Polyclonal Antibody (Bioss Antibodies, USA; dilution 1:200) and Claudin-4: Rabbit polyclonal antibody (Lab Vision Corporation, Fermont, USA/Thermo Scientific); dilution 1:50).** **For HEG1, the rat gut was utilized as a positive control, while for Claudin-4, normal bronchial epithelium was used as a positive control.

### Interpretation and Evaluation of Immunostaining

#### 1. Evaluation of HEG1 immunostaining

On mesothelial cells, the HEG1 expression patterns manifested as diffuse membranous staining; four classes were identified based on the staining intensity groups levels for HEG1: 0 (none), 1 (weak), 2 (moderate), and 3 (strong). About the extension of staining, four groups were established: negative:(0%), one:(<25%), two:(25–50%), and three:(>50%). The number of staining extension categories and staining intensity was added to create a staining score. If the staining score was ≤ 2, a negative value was assigned, and if it was ≥3, a positive value was assigned (6).

#### 2. Evaluation of Claudin-4 immunostaining

Negative staining was considered when membranous staining was shown in less than 10% of cells.

Positive staining was considered when membranous staining was shown in >10% of tumor cells (7).

### Statistical Analysis

The MedCalc Statistical Software version 18.9.1 (MedCalc Software bvba, Ostend, Belgium; http://www.medcalc.org; 2018) was used. All statistical analyses were conducted using SPSS version 22.0 for Windows (IBM Corp., Armonk, NY, USA) and Microsoft Office Excel 2010 for Windows (Microsoft Corp., Redmond, WA, USA). Continuous variables were expressed as the mean, whereas categorical variables were expressed as a number (%) ± SD & median (range). The Shapiro-Wilk test was utilized to verify the normality of continuous data sets. The Mann-Whitney test was used to compare two sets of non-normally distributed data. When comparing the proportion of categorical variables, Fisher’s exact test or Pearson’s Chi-square test was employed. Using the examination of the surgical biopsy specimens as the reference (Gold) standard, sample 2x2 contingency tables were generated to perform diagnostic IHC staining for Claudin-4 and HEG1. For the sensitivity, specificity, accuracy, negative predictive values and positive predictive value, 95% confidence intervals were calculated. There were two sides to every test. Plots were classified as highly statistically significant (S) if they were larger than 0.001 and smaller than 0.05 (HS), and those > 0.05 as statistically insignificant (NS).

## RESULTS

### Immunohistochemical (IHC) results of all studied cases

**Figure 1:**
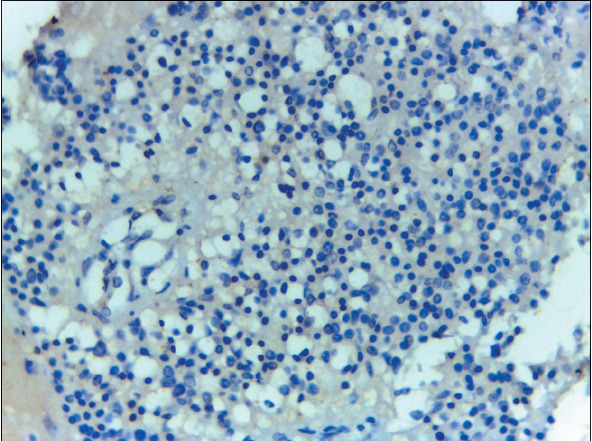
Cell block of pleural effusion showing negativeClaudin-4 ICC staining of RMCs (Claudin-4 X 400, original magnification).

**Figure 2:**
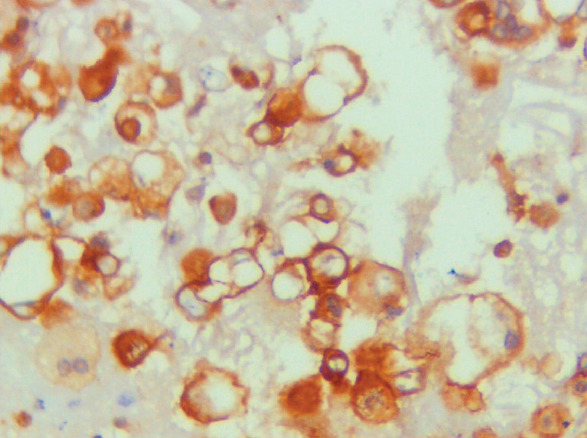
Cell block of pleural effusion showing membranous HEG1 ICC staining of RMCs (HEG1 X400, original magnification).

Whereas 37 out of 64 (57.8%) malignant effusion (lung adenocarcinoma, MPM cases) were Claudin-4 positive, all cases of benign effusion with RMCs were Claudin-4 negative (Figure 1). Of the instances of benign effusion (RMCs), 19/20 (95%) of the cases exhibited HEG1 positivity (Figure 2), and 1/20 (5%) of the patients was negative (Table 1). Of the cases of malignant effusion, 25/64 (39.1%) had HEG1 expression.

**Table 1:** Immunohistochemical expression of Claudin-4 and HEG1 in all studied cases

**Immunohistochemistry staining**	**Benign effusion with RMCs (N=20)**	**Malignant effusion (N=64)**	**Testa**	**p-value (Sig.)**
	**n (%)**	**n (%)**		
**Claudin-4**				
Negative	20 (100)	27 (42.2)	20.665	<0.001
Positive	0 (0)	37 (57.8)		
**HEG1**				
Negative	1 (5)	39 (60.9)	7.6	0.56
Positive	19 (95)	25 (39.1)		
**Claudin-4/HEG1**				
Negative/Negative	1 (5)	2 (3.1)	33.520	<0.001
Negative/Positive	19 (95)	25 (39.1)		
Positive/Negative	0 (0)	37 (57.8)		

**a:** Chi-square test. **p-value <** 0.05 is significant. **Sig.:** Significance.

### Immunohistochemical expression of Claudin-4 and HEG1 in malignant effusion cases

**Figure 3:**
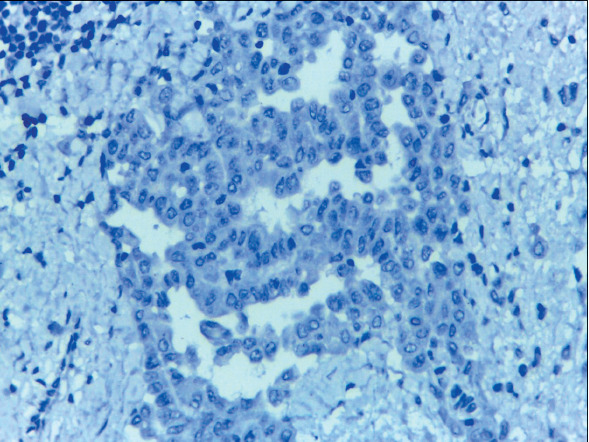
Cell block of pleural effusion of epithelioid mesothelioma, showing negative claudin-4 ICC expression in malignant mesothelial cells (Claudin-4 X 400, original magnification).

**Figure 4:**
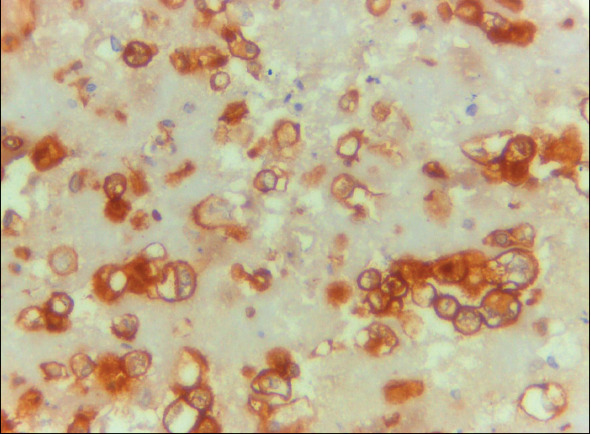
Pleural effusion cell block of adenocarcinoma, the malignant epithelial cells showing strong and diffuse membranous staining for Claudin-4 ICC (Claudin-4 X 400, original magnification).

**Figure 5:**
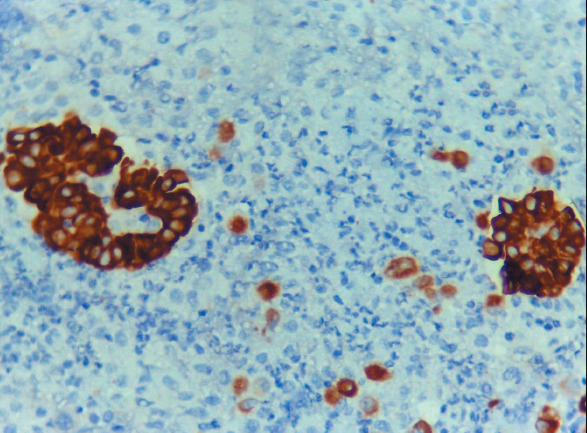
Pleural effusion cell block of adenocarcinoma, the malignant epithelial cells showing strong and diffuse membranous staining for Claudin-4 ICC (Claudin-4 X 100, original magnification).

**Figure 6:**
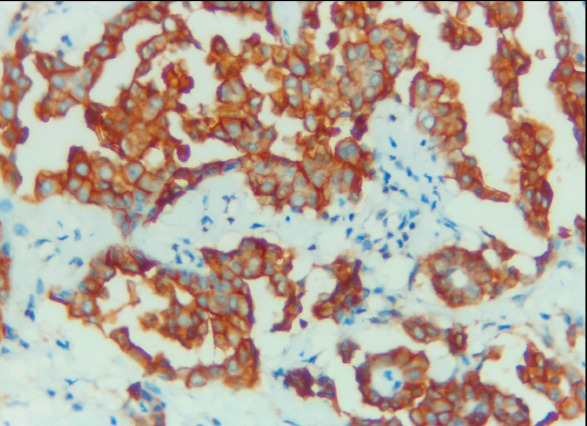
Cell block of pleural effusion of epithelioid mesothelioma, showing diffuse strong membranous staining for HEG1 ICC of malignant mesothelial cells (HEG1 X 400, original magnification).

**Figure 7:**
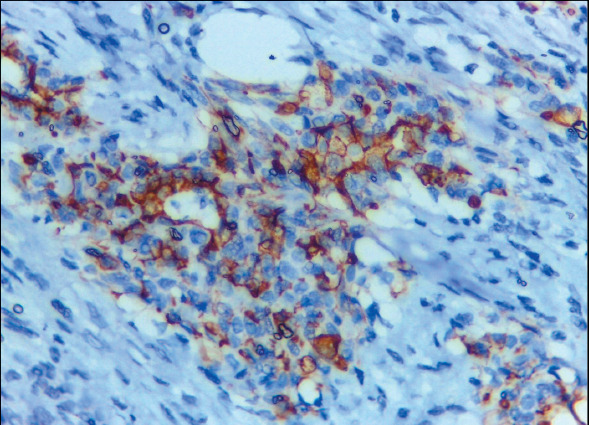
Cell block of pleural effusion of epithelioid mesothelioma, showing moderate membranous staining for HEG1 ICC of malignant mesothelial cells (HEG1 X 400, original magnification).

Claudin-4 was negative in 25/25 (100%) of MPM cases (Figure 3) and positive in 37/39 (94.9%) of lung cancer cases (Figure 4,Figure 5). HEG1 was found to be positive in 24 out of 25 cases (96%) of MPM (Figure 6,Figure 7) and negative in 38 out of 39 cases (97.4%) of lung adenocarcinoma. Table 2 shows that of the cases of lung adenocarcinoma, 37/39 (94.9%) had positive Claudin-4/negative HEG1 (the diagnostic pattern of lung adenocarcinoma) and 24/25 (96%) had negative Claudin-4/positive HEG1 (the diagnostic pattern of MPM).

**Table 2:** Comparison between MPM and lung adenocarcinoma regarding IHC staining for Claudin-4 and HEG1.

**Immunohistochemistry staining**	**MPM (N=25)**	**Lung Adenocarcinoma (N=39)**	**Testa**	**p-value** **(Sig.**)
	**n (%)**	**n (%)**		
**Claudin-4**				
Negative	25 (100)	2 (5.1)	56.220	<0.001
Positive	0 (0)	37 (94.9)		
**HEG1**				
Negative	1 (4)	38 (97.4)	55.874	<0.001
Positive	24 (96)	1 (2.6)		
**Claudin-4/HEG1**				
Negative/Negative	1 (4)	1 (2.6)	57.867	<0.001
Negative/Positive	24 (96)	1 (2.6)		
Positive/Negative	0 (0)	37 (94.9)		

**a:** Chi-square test. **p-value** < 0.05 is significant. **Sig.:** Significance.

### Diagnostic performance of IHC in the differentiation between MPM and lung adenocarcinoma

The results indicate that the diagnostic pattern (-ve Claudin-4/+ve HEG1) has a 96% sensitivity, 97.4% specificity, 96% positive predictive value, 97.4% negative predictive value, and 96.8% overall accuracy in diagnosing MPM (Table 3). The results (Table 4) indicate that the diagnostic pattern for lung adenocarcinoma had the following values: sensitivity (94.8%), specificity (100%), positive predictive value (PPV) (100%), negative predictive value (NPV) (92.5%), and total accuracy (96.8%).

**Table 3:** Diagnostic performance of IHC staining for Claudin-4 and HEG1 in diagnosis of MPM.

**Parameters**	**Claudin-4+HEG1 diagnosis**
True positive	24
False positive	1
True negative	38
False negative	1
Sensitivity (95%CI)	96% (79.648 – 99.899)
Specificity (95%CI)	97.436% (86.524 – 99.935)
PPV (95%CI)	96% (77.586 – 99.403)
NPV (95%CI)	97.436% (84.768 – 99.616)
Accuracy (95%CI)	96.875% (89.163 – 99.619)

**PPV:** Positive Predictive Value. **NPV:** Negative Predictive Value. **CI:** Confidence interval.

**Table 4:** Diagnostic performance of IHC staining for Claudin-4 and HEG1 in diagnosis of lung adenocarcinoma.

**Parameters**	**Claudin-4+HEG1 diagnosis**
True positive	37
False positive	0
True negative	25
False negative	2
Sensitivity (95%CI)	94.872% (82.676 – 99.373)
Specificity (95%CI)	100% (86.281 – 100)
PPV (95%CI)	100%
NPV (95%CI)	92.593% (76.419 – 97.968)
Accuracy (95%CI)	96.875% (89.163 – 99.619)

**PPV:** Positive Predictive Value. **NPV:** Negative Predictive Value. **CI:** Confidence interval.

## DISCUSSION

Cell blocks and cytological smears were generated for the current investigation, and cell block sections made from the sediment of effusion samples were subjected to an IHC procedure. The current study’s HEG-1-stained cell block sections revealed that 96% of MPM cases had positive membranous HEG-1 expression, while only one case of lung adenocarcinoma was positive. Additionally, there was a statistically significant variation in the HEG-1 immunoreactivity between MPM and lung adenocarcinoma. HEG1 is therefore particularly useful in distinguishing lung adenocarcinoma from MPM.

These results are aligned with Itami et al., who reported that in 30 MPM cases and 4 peritoneal mesothelioma cases, HEG1 was positive in all cases (8). Moreover, Hiroshima et al., looked at the cell blocks of 41 cases of malignant mesotheliomas and 54 cases of carcinomas from various sites (26 lung carcinomas, 21 ovarian carcinomas, and 7 other carcinomas) and found that HEG-1 was positive in all of the mesothelioma cases and negative in 48 of the 54 cases of carcinomas, with six cases of ovarian origin that were positive (6).

Another study by Tsuji et al., discovered that 108 out of 112 (96%) mesotheliomas, including biphasic and epithelioid had positive membrane staining, whereas none of the 98 pulmonary carcinomas reacted to the SKM9-2 antigen (monoclonal antibody against sialylated HEG1) (9).

In a study by Naso et al., where tissue microarrays were used, positive HEG1 membrane IHC staining was observed in 94% of epithelioid mesotheliomas and negative membranous HEG1 IHC staining in all 167 (100%) NSCLC (73 pulmonary adenocarcinomas, 60 pulmonary SCCs, 21 pulmonary sarcomatoid carcinomas and 13 pulmonary large cell carcinomas) with the exception of one case of focal weak membrane staining in lung adenocarcinoma (scored as negative). Of the 73 pulmonary adenocarcinomas, 3 (4%) had weak but diffuse HEG1 cytoplasmic staining. In these cancer samples, the cytoplasmic staining was counted as negative because the epithelioid mesotheliomas displayed membranous staining rather than cytoplasmic staining. The twenty-one lung sarcomatoid carcinomas did not exhibit any cytoplasmic HEG1 staining. For mesothelioma as opposed to lung adenocarcinoma, they concluded that membranous HEG1 IHC staining exhibited 100% specificity (10).

A recent study summarizing the mesothelioma positive rates indicated that the SKM9-2 antigen (sialylated HEG1) was more useful than conventional markers (calretinin, WT-1, and podoplanin) in detecting the major histological forms of malignant mesothelioma (epithelioid-type) markers: SKM9-2, 97%; WT-1, 81%; podoplanin, 84%; calretinin, 92% (11).

Data for HEG1, derived from four laboratories, showed membrane staining in only 393 of 434 (91%) epithelioid/biphasic mesotheliomas in contrast to previous study that reported 97% of postivity and this may be due to dissimilar cohort number or different methods of positivity interpretation. On the other hand, only one case of 360 (0.3%) NSCLC showed HEG1 positivity (sensitivity 91%, specificity 99.7%) (12).

In 95% of RMCs with benign effusions, cell block sections from this study demonstrated positive membranous HEG-1 immunoexpression; in contrast, just one case of lung adenocarcinoma displayed similar immunoexpression. HEG1 is therefore particularly useful in distinguishing lung adenocarcinomas from RMCs.

These results were compatible with a small variation with those of Hiroshima et al., who looked at the cell blocks of 26 benign cases with RMCs and 54 carcinomas from various sites (26 of which were lung carcinomas), and they found that, out of the 54 carcinoma cases, HEG-1 was negative in 48 of them, with the exception of 6 instances with ovarian origin that were positive. Only 76.9% (20/26) of the cases involving RMCs were positive; the majority of the solitary RMCs displayed membrane staining, while some of them also had mild cytoplasmic staining. This discrepancy appears to be the result of various approaches of interpreting marker positive cases (6).

In a study by Naso et al., with regard to benign patients, only 88% of reactive epithelial mesothelial proliferations and 34% of reactive spindle cell mesothelial proliferations were HEG1 positive. HEG1 membranous IHC expression was negative in all 167 (100%) of NSCLC. On the other hand, reactive spindle cell proliferation typically showed faint, localized staining (10).

According to this study, 96% of MPM cases and 95% of cases involving RMCs with benign effusions had positive membranous HEG-1 immunoexpression. As a result, we believed that HEG1 is incapable of differentiating between mesothelioma and RMC instances. Our outcome agreed with the findings of Naso et al., and Hiroshima et al. (6,10).

In addition, we discovered that HEG-1 had the following MPM diagnostic parameters: sensitivity (96%) and specificity (97.4%), accuracy (96.8%), positive predictive value (97.43%), and negative predictive value (96%).

Cytoplasmic-membranous staining for HEG1 has been shown, based on two therapeutically focused investigations, to be 92–99% sensitive and 83–99% specific for the mesothelial lineage overall. HEG1 outperformed calretinin, D2-40, and WT-1 IHC in terms of sensitivity and specificity in both investigations (9,13).

Moreover, Naso et al., found that the remarkably excellent specificity of HEG1 IHC (100%) and comparable sensitivity (94%) for epithelioid mesothelioma may mean that additional mesothelial markers are not necessary to establish a diagnosis, in contrast to NSCLC (10).

The most frequent causes of metastatic MPE are lung, breast, lymphoma, ovarian, and stomach cancer, in that order of frequency. Eighty percent of MPE are these malignancies (14).

Cytologic features like occasional signet ring cell shape, cytoplasmic vacuoles, and larger nucleated cells in singles or clusters help diagnose adenocarcinoma. Adenocarcinoma cells show positive staining for TTF1, Napsin A, monoclonal CEA, and CK-7. When needed, IHC stains on pleural fluid are used to distinguish mesothelial cells from cancer (15). Few single markers have shown high sensitivity and specificity. Various IHC marker combinations have been suggested to differentiate MPM from metastatic cancers and benign mesothelial growths (16). Current markers include mesothelial (calretinin, CK5/6, mesothelin, WT-1, D2-40) and epithelial (CEA, Ber-Ep4, B72.3, MOC-31) markers. Due to the low sensitivity and specificity of single markers, IHC panels with multiple markers are often necessary but are costly and impractical for frequent use in resource-limited centers with high workloads. The search for highly accurate and affordable markers continues (17).

Claudin-4, a tight junction-associated protein that is indicative of epithelial development, is produced by almost all carcinomas (18).

In the current study, all 25 cases of MPM and 100% of benign effusions with RMCs had negative Claudin-4 immunoexpression. Of the 39 patients with lung adenocarcinomas, 37 (94.9%) had positive membrane reactivity. On the other hand, there was a statistically significant difference in the immunoexpression of Claudin-4 between lung cancer, benign effusion with RMCs, and MPM (p value <0.001). As a result, Claudin-4 is a helpful marker for differentiating reactive or malignant mesothelial cells from lung cancer.

The current study’s findings were consistent with Patel et al., who evaluated the expression of Claudin-4 in 58 pleural effusion cell block sections, comprising 10 benign effusions with RMCs, 8 instances of MPM, 40 lung adenocarcinomas. They discovered that all mesotheliomas and benign effusions with RMCs tested negative for Claudin-4, but 100% of adenocarcinomas tested positive. They concluded that MPM or RMCs in pleural effusions may be successfully distinguished from lung adenocarcinoma using Claudin-4 IHC (19).

A previous study by Vojtek et al., has reported similar findings, assessing Claudin-4 expression in cell-block sections of 284 effusions, comprising 49 benign reactive lesions, 63 MPMs, and 172 metastatic tumors with multiple sources and cell differentiation. They discovered that all benign effusion patients with RMCs tested negative for Claudin-4, while only one mesothelioma case (1.6%) and 131/137 (95.6%) adenocarcinoma cases (including all 34 cases of lung adenocarcinoma) tested positive (20).

In another study by Bernardi et al., Claudin-4 expression was assessed in 49 MPM on cytology with 43 matched biopsies, 49 normal/reactive mesothelial proliferations, and 49 pleural metastatic carcinomas from different primaries with 21 matched pleural biopsies. The results demonstrated that while all metastatic carcinomas showed membrane Claudin-4 staining, all reactive and malignant mesothelial cells tested negative for the protein (16).

In a study by Najjar et al., 134 of the 143 carcinoma specimens were positive for Claudin-4, but all mesotheliomas (0/39) and benign effusions with RMCs (0/47) were negative (21).

Furthermore, Kim et al. found that there was mild membrane staining in 27.5% of reactive mesothelial instances. Claudin-4 IHC staining was seen in all 194 (100%) cases of metastatic adenocarcinoma cells and 11 (27.5%) out of 40 cases of RMCs (22).

Furthermore, we discovered that the Claudin-4 sensitivity was 94.8%, specificity was 100%, negative predictive value was (92.5%), positive predictive value was (100%), and accuracy was 96.8% when it came to the diagnosis of lung adenocarcinoma.

Eccher et al., stated that, although being a relatively new marker, Claudin-4 has shown up to 92% to 100% sensitivity and 94% to 100% specificity for epithelial cells, and there is increasing consensus that this epithelial marker could be the most effective for use in diagnostic applications (23).

Furthermore, in line with our findings, Vojtek et al. have discovered that Claudin-4 has the following values: 95.6% sensitivity, 99.1% specificity, 94.9% negative predictive value, and 99.2% positive predictive value (20).

When it came to differentiating MPM from metastatic cancer, Claudin-4 IHC staining demonstrated 100% sensitivity, specificity, PPV, NPV, and DA (diagnostic accuracy), with a statistically significant p value (p<0.00001) (16).

Recently, Najjar et al. concluded that in comparison to MOC-31 and Ber-EP4, Claudin-4 can be employed as a single marker for cancer with high sensitivity (93.7%) and higher specificity (100%). A positive result for Claudin-4 rules out the mesothelial lineage (21).

Reports assessing Claudin-4 in non-small cell lung cancer revealed mild positive results in 469 of 502 (93%) carcinomas and five of 463 (1.0%) epithelioid/biphasic mesotheliomas (sensitivity 93%, specificity 98.9%) (12).

However, Lee et al. concluded that the claudin-4 reactivity was 100% sensitive and only 72.5% specific, with a positive predictive value of 94.6% and a negative predictive value of 100% (24). Churg and Naso have recently hypothesized that the use of only two immunostains usually could enable the highly accurate differentiation between epithelioid/biphasic mesotheliomas from NSCLC carcinomas using HEG1 and Claudin-4 IHC labelling in combination (12). Although more information is needed on HEG1 SKM9-2 staining of carcinomas other than non-small cell lung cancer (NSCLC), this combination is probably also useful for carcinomas from most other sites. This method would make the diagnosis of mesothelioma much easier.

## CONCLUSION

HEG1 and Claudin-4 IHC staining together are particularly useful for differentiating reactive or malignant mesothelial cells from adenocarcinoma in pleural effusion.

## Conflict of Interest

The authors have no conflicts of interest to declare.

## Financial Disclosure

none.
